# External quality assurance of chest X-ray interpretation to strengthen diagnosis of childhood TB

**DOI:** 10.5588/ijtldopen.24.0328

**Published:** 2024-10-01

**Authors:** B.F. Melingui, E. Leroy-Terquem, J.V. Taguebue, T.C. Eap, L. Borand, C. Khosa, R. Moh, J. Mwanga-Amumpaire, S. Beneteau, M.T. Eang, I. Manhiça, A. Mustapha, O. Marcy, E. Wobudeya, P.Y. Norval, M. Bonnet

**Affiliations:** ^1^ Institut de Recherche pour le développement (IRD), University of Montpellier, Montpellier, France;; ^2^ François Quesnay Hospital, International Pulmonology Support, Mantes-la-Jolie, France;; 3 Mother and Child Center, Chantal Biya Foundation, Yaoundé, Cameroon;; 4 National Tuberculosis Programme, Ministry of Health, Phnom Penh, Cambodia;; 5 Institut Pasteur of Cambodia, Epidemiology and Public Health Unit, Clinical Research Group, Phnom Penh, Cambodia;; 6 Center for Tuberculosis Research, Division of Infectious Diseases, Johns Hopkins University School of Medicine, Baltimore, MD, USA;; 7 National Institute of Health, Marracuene, Mozambique;; 8 Programme d’Action Coordonné - Cote d’Ivoire, Treichville University Hospital, Abidjan, Côte d'Ivoire;; 9 Epicentre Mbarara Research Centre, Mbarara, Uganda;; 10 National Center for Tuberculosis and Leprosy, Ministry of Health, Phnom Penh, Cambodia;; ^11^ National Tuberculosis Control Programme, Ministry of Health, Maputo, Mozambique;; ^12^ Ola During Children’s Hospital, Freetown, Sierra Leone;; 13 University of Bordeaux, Institut national de la santé et de la recherche médicale, Unité mixte de Recherche 1219, IRD, Bordeaux, France;; 14 Makerere University–Johns Hopkins University Research Collaboration Care, Kampala, Uganda;; 15 Technical Assistance for Management, Paris, France.

**Keywords:** external quality assurance, tuberculosis, diagnosis, children, chest X-ray, limited-resources countries

## Abstract

**BACKGROUND:**

Chest X-ray (CXR) misinterpretation negatively affects the accuracy of childhood TB diagnosis. External quality assurance (EQA) could strengthen CXR reading skills. We assessed the uptake, performance and challenges of an EQA of CXR interpretation within the childhood TB-Speed decentralisation study in six resource-limited countries.

**METHODS:**

Every quarter, TB suggestive or unreadable CXRs and 10% of remaining CXRs from children with presumptive TB were selected for blind re-reading by national re-readers. The proportion of CXRs selected for EQA and re-read assessed the uptake. The performance was assessed by the proportion of discordant interpretations and the sensitivity and specificity of clinicians’ vs re-readers’ interpretations. Challenges were retrieved from country reports.

**RESULTS:**

Of 513 eligible CXRs, 309 (60.8%) were selected for EQA and 278/309 (90.0%) re-read. The proportion of discordant interpretation was between 13/48 (27%) in Sierra Leone and 7/13 (53.8%) in Cote d’Ivoire during the first EQA and decreased after the EQAs periods in 3/5 countries. Clinician sensitivity reached 100% in all countries over EQA. Specificity ranged between 13% in Sierra Leone and 65% in Cambodia (first EQA) and increased in 4/5 countries after the EQA periods. CXR transfer and re-readers’ workload were the main challenges.

**CONCLUSION:**

EQA can enhance CXR interpretation for childhood TB diagnosis, provided operational challenges are overcome.

## RÉSUMÉ

**CONTEXTE:**

Les erreurs d'interprétation du radio thoracique (CXR, pour l’anglais « *chest X-ray* ») compromettent la précision du diagnostic de la TB chez les enfants. La mise en place d'une assurance qualité externe (EQA, pour l’anglais « *external quality assurance* ») pourrait améliorer les compétences en lecture des CXR. Nous avons examiné l'adoption, l'efficacité et les obstacles liés à une EQA pour l'interprétation des CXR dans le cadre de l'étude de décentralisation *Child TB-Speed*, menée dans six pays à ressources limitées.

**MÉTHODES:**

Chaque trimestre, les CXRs évocatrices de TB ou illisibles, ainsi que 10% des autres CXRs d'enfants atteints de TB présumés, ont été choisies pour une relecture en aveugle par des experts nationaux. La proportion de CXRs sélectionnées pour EQA et relues a permis d'analyser le taux de participation. La performance a été mesurée en fonction du nombre d'interprétations discordantes, ainsi que de la sensibilité et de la spécificité des interprétations des cliniciens comparées à celles des relecteurs. Les contestations ont été extraites des rapports nationaux.

**RÉSULTATS:**

Parmi les 513 CXR éligibles, 309 (60,8%) ont été retenus pour l'EQA, et 278 sur 309 (90,0%) ont été réévalués. La proportion d'interprétations discordantes variait de 13 sur 48 (27%) en Sierra Leone à 7 sur 13 (53,8%) en Côte d'Ivoire lors de la première EQA, et a diminué après les périodes d'EQA dans trois des cinq pays. La sensibilité des cliniciens a atteint 100% dans tous les pays durant l'EQA. La spécificité a fluctué entre 13% en Sierra Leone et 65% au Cambodge (première EQA), et a augmenté dans quatre des cinq pays après les périodes d'EQA. Le transfert des CXRs ainsi que la charge de travail des relecteurs ont représenté les principaux défis rencontrés.

**CONCLUSION:**

L'EQA a le potentiel d'optimiser l'analyse des CXRs pour le diagnostic de la TB chez l'enfant, sous réserve de surmonter les obstacles opérationnels.

Most children with TB are diagnosed based on suggestive clinical and radiological signs.^1,2^ Chest X-ray (CXR) can identify features highly suggestive of TB, such as mediastinal adenopathies, cavities, miliary patterns and features that are less specific but common in children with TB, such as alveolar opacity and pleural effusion.^3^ CXR interpretation is challenging in young children, notably for TB diagnosis. The persistence of thymus in children less than 2 years can be mistaken with mediastinal adenopathies, and immunosuppressed children are more likely to present with atypical radiological features of TB.^3,4^

CXR interpretation for childhood TB detection is known to have a low inter-reader reproducibility, and its performance highly depends on readers’ experience.^5–7^ In high TB incidence and resource-limited countries, there is also a lack of training in CXR interpretation for diagnosis of childhood TB, especially at low healthcare level.^4,8^ Several training materials have been recently developed to improve reading skills.^9,10^ Access to digital radiography can enhance the quality of CXR by eliminating problems of lack or use of expired reagents and films common with analogue radiography in limited-resource settings.^11^

So far, little attention has been given to quality assurance of CXR interpretation as a means of continuous skill improvement after training. Other TB diagnostic tests that are highly observer-dependent, such as smear microscopy, benefit from external quality assurance (EQA).^12^ Based on experience in Myanmar, some authors suggest EQA for CXR interpretation as a tool to be used during supervision visits with a discussion of discordant interpretations.^13^

As part of a large international operational study aiming to assess decentralised models of childhood TB diagnosis, we sought to determine the uptake, performance and challenges associated with implementing an EQA for CXR interpretation at low levels of healthcare facilities.

## METHODS

### Study design and settings

The research used a mixed methodology. It was embedded in the TB-Speed Decentralisation study that assessed the effect on paediatric TB case detection by implementing a comprehensive diagnostic package at district hospital (DH) and primary health centres (PHC) levels in Cameroon, Ivory Coast, Mozambique, Uganda, Sierra Leone, Zambia and Cambodia from September 2020 to September 2021. The package included 1) symptomatic screening of all sick children below 15 years attending the health facility to identify children with presumptive TB and, in those identified with presumptive TB, 2) specimen collection and Xpert^®^ MTB/RIF Ultra testing on nasopharyngeal aspirate and stool or sputum sample, 3) clinical evaluation, and 4) CXR (all children with presumptive TB at DH and only children with persisting symptoms after 1 week at PHC). Two districts were selected in each country, with one DH and four PHCs per district.

### Radiography equipment and training

Radiology departments from DHs were equipped with digital radiography plates (Agfa DR14eC; AGFA, Rueil-Malmaison, France). Radiographers were trained on how to convert Digital Imaging and Communication in Medicine (DICOM) images format into Joint Photographic Experts Group (JPEG) format and to upload them on the international FTPS (File Transfer Protocol Secure) server at the University of Bordeaux, Bordeaux, France).

For all children in whom a CXR was indicated, standard posteroanterior was performed in children aged ≥5 years or anteroposterior, and lateral view (in children below 5 years of age) was performed.

Clinicians and radiographers underwent a 1.5-day training on recognising good quality, normal and TB-suggestive CXRs. A quarter of them (45/191) had CXR reading experience.^10^ The presence of at least one of six radiological features (enlarged lymph nodes, alveolar opacity of lung tissue, airway compression, miliary pattern, cavities, pleural or pericardial effusion) defined a TB suggestive CXR.^10^ DH and PHC CXR readers were medical doctors, clinical officers or nurses. Clinicians downloaded CXRs from the server on a tablet for interpretation and filled out an electronic Case Report Form (e-CRF) connected to a REDCap database (Vanderbilt University, Nashville, TN, USA) to report their interpretation (Supplementary Figure S1).

### External quality assurance procedure

Inspired by the EQA for smear-microscopy,^12^ we proposed a similar approach for CXR interpretation. The purpose of the EQA was to ensure and maintain the quality of clinicians’ CXR interpretation for clinical TB diagnosis and identify clinicians who may require refresher training. Discordant CXR interpretations between clinicians and re-readers were used as discussion points to strengthen clinicians’ skills during clinical mentoring visits. In each country, a radiologist or paediatrician with paediatric TB experience was identified as a national re-reader and read a selection of CXR using the same criteria as used by clinicians to define a CXR suggestive of TB (at least one of the six suggestive radiological features).

The selection of CXR consisted of a random selection of 10% of CXRs defined as not suggestive of TB, and all CXRs reported as TB suggestive or unreadable by clinicians over quarterly periods. This selection was generated by the international data manager at the University of Bordeaux. National re-readers were trained to download selected CXRs from the server on a tablet for interpretation and reported their findings using the same e-CRF as clinicians. Re-readers were blinded to the clinician’s interpretation and the children’s clinical information. Four EQA rounds were expected in each country over the 12-month study intervention period, except in Mozambique (3 EQAs over a 9-month intervention period). Country data managers exported the clinician’s and national re-reader’s interpretations to prepare the EQA reports (Supplementary Table S1). From this report, the list of CXRs with discordant interpretation between clinicians and national re-readers was shared with the clinical mentor for discussion with the clinician at the scheduled mentoring visit (monthly in the first quarter, then quarterly).

A radiological expert based in France supervised the EQA with the re-reading of CXRs to assess the performance of the national re-readers and remote meetings gathering national re-readers, study country principal investigator and clinical mentors after each EQA to discuss clinicians’ performance and EQA challenges.

### Outcomes and data sources

Outcomes of interest were EQA uptake and performance and the challenges faced. Uptake outcomes retrieved from the clinical database included the number of EQAs performed and the proportion of 1) CXR selected for EQA among all CXRs eligible to EQA based on pre-defined criteria; 2) the proportion of CXRs re-read among selected CXRs. EQA performance outcomes used data from the clinician’s CXR interpretation in the clinical database and the national re-reader’s interpretation from an MS Excel EQA database (Microsoft, Redmond, WA, USA). Outcomes included the proportion of discordant CXRs’ interpretation between clinicians and re-readers (TB suggestive vs non-suggestive) and the sensitivity and specificity of the clinician’s interpretation against the re-reader’s interpretation. Sensitivity was the proportion of TB suggestive CXRs as per national re-reader interpretation that was correctly identified by the clinician, and specificity was the proportion of non-TB suggestive CXRs (normal or abnormal without TB suggestive patterns) as per national re-reader interpretation that was correctly identified by the clinician. The proportion of discordant interpretations was also assessed for each TB suggestive feature. The performance outcome of the national re-reader was assessed by the agreement between national and international readers’ interpretations to identify TB suggestive CXR.

EQA challenges were retrieved from the EQA supervisory meeting reports, including technical challenges (e.g., image transfer difficulties) and operational challenges (e.g., clinicians’ reporting delays and re-readers’ workload).

### Data analysis

The proportion of discordances, sensitivity, specificity and their 95% confidence interval (CI) were calculated per country and EQA. We calculated the prevalence-adjusted and bias-adjusted kappa (PABAK) to assess the inter-reader agreement between national and international re-readers to take into account the low proportion of CXRs suggestive of TB and used Landis et Koch classification to characterise the strength of agreement.^14^ Data were analysed using R software v4.2.1 (R Foundation for Statistical Computing, Vienna, Austria). Challenges extracted from meeting reports as free text were grouped into global deductive themes.

### Ethics statement

The TB-Speed decentralisation study was approved by the national ethics committees of each country, the WHO Ethical Review Board, and the Institut national de la santé et de la recherche médicale Ethics Evaluation Committee, Paris, France.

## RESULTS

### EQA uptake

Of 1,780 CXRs performed, 513 (29%) fitted criteria for EQA, 309/513 (61%) were selected for EQA, and 278/309 (90%) were interpreted by national re-readers. In four out of six countries, the proportion of CXRs selected was less than 80% (Table 1). Only two countries performed the number of EQAs as planned.

**Table 1.** Uptake of the CXR external quality assurance per country and overall.

Country	EQA cycles *n*	CXR performed *n*	CXR not readable *n* (%)	CXR not suggestive of TB *n* (%)	CXR suggestive of TB *n* (%)	CXR eligible to EQA n*	CXRs selected for EQA *n* (%)	CXR read by national re-reader *n* (%)
Cambodia	03	209	0 (0)	197 (94.2)	12 (5.7)	32	32 (100)	32 (100)
Cameroon	04	523	0 (0)	445 (85.0)	78 (14.9)	123	57 (46.3)	52 (91.2)
Côte d’Ivoire	03	231	0 (0)	153 (66.2)	78 (33.7)	93	80 (86.1)	45 (56.2)
Mozambique	02	300	0 (0)	246 (82.0)	54 (18.0)	78	35 (44.8)	35 (100)
Sierra Leone	03	338	0 (0)	222 (65.6)	116 (34.3)	138	78 (56.1)	75 (96.1)
Uganda	04	179	2 (1.1)	144 (80.4)	33 (18.4)	49	27 (55.1)	39 (100)^†^
Total	19	1,780	2 (0.1)	1,407 (79.1)	371 (20.8)	513	309 (60.2)	278

* All X-rays suggestive of TB, all unreadable X-rays and 10% of X-rays non-suggestive of TB. ^†^ At the special request of clinicians, 12 CXRs in Uganda were reviewed by the national re-reader and included in the analysis.

CXR = chest-X-ray; EQA = external quality assurance.

### EQA performance

The proportion of discordant CXRs interpretation ranged between 27% (Sierra Leone) and 54% (Cote d’Ivoire) during the first EQA, with a decrease after the EQA period in 3/5 countries (Table 2, Supplementary Table S2). The sensitivity of clinicians’ CXR interpretation was 100% across all EQAs in Cambodia, Cameroon and Sierra Leone and increased to 100% during the last EQAs in Cote d’Ivoire and Uganda. It decreased from 71% to 50% in Mozambique. The specificity ranged between 13% in Sierra Leone and 65% in Cambodia during the first EQA and increased in four countries (Cote d’Ivoire, Sierra Leone, Mozambique and Uganda) over EQAs (Figure, Supplementary Table S3).

**Table 2.** Proportion of discordant chest X-ray interpretation between clinician and national e-reader over EQA per country.

Round	Cambodia		Cameroun		Cote d’ivoire		Mozambique		Sierra Leone		Uganda	
	n*	Proportion of total number of discordances *n* (%)	n*	Proportion of total number of discordances *n* (%)	n*	Proportion of total number of discordances *n* (%)	n*	Proportion of total number of discordances *n* (%)	n*	Proportion of total number of discordances *n* (%)	n*	Proportion of total number of discordances *n* (%)
EQA 1	18	6 (33.3)	12	4 (33.3)	13	7 (53.8)	26	9 (34.6)	48	13 (27.0)	10	5 (50.0)
EQA 2	7	0 (0.0)	11	0 (0.0)	8	0 (0.0)	9	5 (55.5)	13	3 (23.0)	5	2 (40.0)
EQA 3	7	2 (28.5)	13	8 (61.5)	24	0 (0.0)			14	1 (7.1)	8	0 (0.0)
EQA 4			16	4 (25.5)							16	0 (0.0)

* Number of chest X-rays examined.

EQA = external quality assurance.

**Figure.**
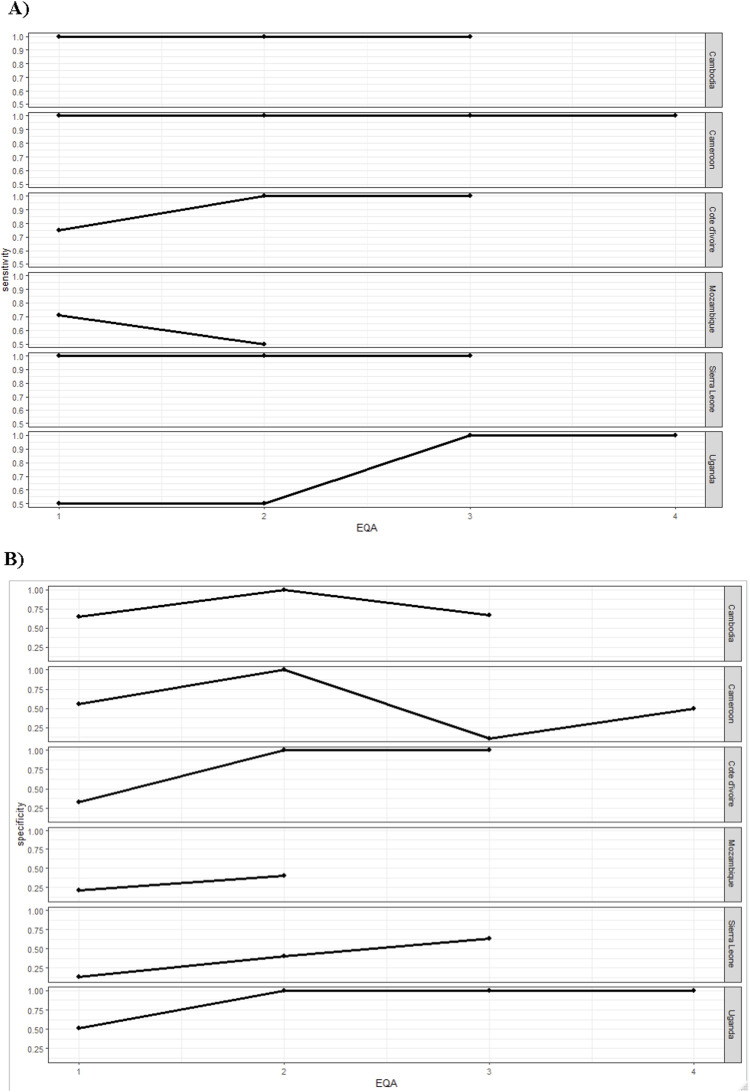
**A)** Sensitivity and **B)** specificity of the chest X-ray interpretation by clinicians against national re-reader’s interpretation over EQA per country. EQA = external quality assurance.

The PABAK between national and international re-readers ranged between 0.35 in Uganda and 1.0 in Cameroon and the Ivory Coast (Table 3).

**Table 3.** Inter-reader agreement of chest X-ray interpretation between national re-reader and international re-reader.

	Cambodia		Cameroon		Ivory Coast		Mozambique		Sierra Leone		Uganda	
	Suggestive of TB	Not suggestive of TB	Suggestive of TB	Not suggestive of TB	Suggestive of TB	Not suggestive of TB	Suggestive of TB	Not suggestive of TB	Suggestive of TB	Not suggestive of TB	Suggestive of TB	Not suggestive of TB
National re-reader	3	28	13	25	15	21	11	14	16	17	19	15
International re-reader												
Suggestive of TB	2	2	13	0	15	0	7	2	14	1	10	2
Not suggestive of TB	1	26	0	25	0	21	4	12	2	16	9	13
PABAK (95% CI)	0.80 (0.48–0.95)		1 (0.81–1)		1 (0.80–1)		0.52 (0.09–0.81)		0.81 (0.51–0.96)		0.35 (0.01–0.65)	

PABAK = prevalence-adjusted and bias-adjusted kappa; CI = confidence interval.

### EQA challenges

Technical challenges included the difficulty in depositing images in the FTPS platform and the complexity of converting the CXR format from DICOM to JPEG, which resulted in delays for CXR transfers and missing CXRs for EQA. Operational challenges included delays in clinicians entering the CXR interpretations into the e-CRF and difficulties retaining re-readers to interpret selected CXRs in some countries despite the study subsidising CXR reading. This latter difficulty was explained by the workload, other priorities, fatigue or lack of motivation on the part of clinicians or radiologists to carry out EQA tasks as opposed to clinical or research tasks and lack of understanding of the potential benefits of the EQA in enhancing clinicians’ skills. Clinical mentors identified the lack of formal refresher training on performance-based CXR interpretation and the few discordant CXR interpretations available for discussion for some EQA rounds as challenges to strengthening clinicians’ CXR reading skills.

## DISCUSSION

A CXR interpretation EQA inspired by the approach to smear microscopy, implemented in the context of operational research in six high TB incidence, resources-limited countries, combined with the review of discordant CXRs during mentoring visits, contributed to improved CXR interpretation by clinicians at DH and PHC. The uptake and performance of EQA varied across countries. Overall, the uptake of the EQA was lower than expected, mainly due to the technical difficulties with image transfer and operational challenges related to human resources workload and priorities, as well as a need to understand the potential benefits of the EQA.

The sensitivity of clinicians to correctly identify a CXR suggestive of TB was high in most countries, resulting in a few missing TB-suggestive CXRs. On the other hand, the specificity was low (<80%) in all countries and very low (<60%) in three countries, raising the issue of over-interpretation of CXR as suggestive of TB and potentially over-diagnosis of TB. However, we observed a trend of improvement in specificity over the EQAs in four countries. This low specificity may highlight the lack of confidence of clinicians to conclude that a CXR is normal or abnormal but non-suggestive of TB and supports the need for more training and mentoring to interpret non-TB suggestive or normal CXR.

To our knowledge, this is the first published experience of EQA for CXR interpretation in the context of TB diagnosis. A study conducted in referral hospitals in Burkina Faso, Cambodia, Cameroon and Vietnam among HIV-positive children with presumptive TB reported sensitivity and specificity of two radiologists and one paediatrician of respectively 71.4% and 50.0%.^15^ In another study conducted in South Africa in children with presumptive TB, clinicians’ CXR interpretation had a 67.0% sensitivity and 48.0% specificity to detect paediatric TB.^8^ These diagnostic accuracy studies used more robust radiological reference standards based on CXR interpretation by at least two blinded independent readers and different classifications of TB suggestive CXR than our study. In our study, sensitivity and specificity results were used to monitor clinicians’ ability to correctly identify a CXR suggestive of TB and strengthen their reading skills. It was evaluated to generate data on the effectiveness and feasibility of the CXR EQA for future programmatic use. In our study, the level of agreement between national re-readers and the international supervisor was substantial in four countries, moderate in one and fair in one.

The quality of CXR is also important as it can affect the interpretation and was assessed through the EQA.^16^ It is also important to set up an EQA system to facilitate the transfer of images. However, despite this technology being installed in each site by the TB-Speed project, the uptake of the EQA was low. This was explained by technical difficulties in uploading images to the server and converting them into the appropriate format for reading, as well as delays by radiographers in uploading images or clinicians in entering their interpretation into e-CRF, often due to poor internet connection. Because of this, the quarterly selection of CXR for the EQA, which was based on the images uploaded and CXR interpretation reports entered into e-CRF, was incomplete, notably in Cameroon and Sierra Leone. Establishing a simple, reliable and durable system for transferring images is crucial for the success and acceptance of such CXR EQA. This would require significant investment by programmes.

Another reported challenge was identifying experienced re-readers and securing their commitment over time. Radiologists or paediatricians of referral centres are often solicited for extra-clinical activities (research, management, programmatic) and may not be interested in long-term quality control activities. Therefore, establishing a pool of re-readers and ensuring gratification is crucial. Unlike smear microscopy, which is fully integrated into the activities of the national tuberculosis control programme and fully subsidised by the programme, radiography is not the responsibility of the programme since it is used for many indications other than tuberculosis. As a result, the EQA system for TB diagnosis may be difficult to implement. Programs, therefore, need to be convinced of the value of CXR EQA in contributing to the good quality of TB diagnosis. Unfortunately, no clear recommendation from WHO for such CXR EQA exists.

The development of computer-aided detection (CAD), which is still under development for the diagnosis of childhood TB, is an alternative to the interpretation of CXR by clinicians who could question the rationale of setting up CXR EQA. CAD will undoubtedly play a critical role in the use of CXR for diagnosis of paediatric TB in the coming years, especially for settings with less experienced and skilled staff,^17^ but it is unlikely to completely replace clinician interpretation of CXR.

This study has limitations: 1) The EQA did not consider abnormal non-TB suggestive CXR. This is because the training was developed for healthcare workers with limited CXR reading experience and focused mainly on recognising the six TB suggestive patterns to identify a TB suggestive CXR. This is likely to bias the clinician’s reading towards TB suggestive. There was limited emphasis on the recognition of abnormal non-TB suggestive and normal CXRs; 2) the use of single reading by one national re-reader is not the best reference standard as compared with double independent readings but was a more sustainable option for future implementation of the EQA. Nevertheless, it is important to control the CXR interpretation of national re-readers to ensure the reliability of the EQA results over time; 3) The small number of CXRs included in each EQA round is a limitation resulting in low precision of the accuracy and concordance results.

## CONCLUSION

In conclusion, external quality assurance of CXR interpretation should be considered as a useful complementary tool to implement and monitor training on CXR interpretation using the material recently developed by the TB-Speed Project and the International Union Against Tuberculosis and Lung Disease, and should be accompanied by a process of support for clinicians through clinical mentoring and refresher training.^18^ Implementing the EQA requires a major political commitment to secure the resources needed to mobilise the technical and clinical expertise required for this type of programme to run smoothly and be sustainable.

## Supplementary Material


