# Supplementary material for: External quality assurance of chest X-ray interpretation to strengthen diagnosis of childhood TB
Source: IJTLD Open. 2024 Oct 1;1(10):449–55. doi: 10.5588/ijtldopen.24.0328 (PMC11467854; doi:10.5588/ijtldopen.24.0328)
Supplement: Supplementary file 1 [file ijtldopen24-0328_supplementarydata1.pdf]

http://dx.doi.org/10.5588/ijtldopen.24.0328

External quality assurance of chest X-ray interpretation to strengthen diagnosis of childhood TB

Supplementary Figure S1: Chest x-ray form for national re-reader

Patient ID (generated)

Date of Chest X-ray (dd/mm/yyyy) _____/______/ ________

Quality of chest X-ray

☐ Acceptable ☐ Unacceptable

Presence of CXR features

Alveolar opacity	☐ No	☐ Yes
Miliary	☐ No	☐ Yes
Cavitation	☐ No	☐ Yes
Enlarged lymph nodes	☐ No	☐ Yes
Airway compression	☐ No	☐ Yes
Pleural / Pericardial effusion	☐ No	☐ Yes

Interpretation

☐ Not suggestive of TB ☐ Suggestive of TB ☐ Not readable

Supplementary Table S1: Example of EQA report of national re-reader

			Quality		Alveolar opacity		Miliary		Cavitation		Enlarged lymph nodes		Airway compression		Pleural/pericardial effusion		Result		Features status
Patient ID	CXR Date	Site	clinician	National re-reader	clinician	National re-reader	clinician	National re-reader	clinician	National re-reader	clinician	National re-reader	clinician	National re-reader	clinician	National re-reader	clinician	National re-reader	
1DXXX0001	01/07/2020	Bafia	Acceptable	Acceptable	Yes	Yes	No	No	No	No	No	No	No	No	No	No	Suggestive of TB	Suggestive of TB	Concordant
1DXXX0002	05/08/2020	Kambuga	**Acceptable**	**Acceptable**	**No**	**Yes**	No	No	No	No	No	No	No	No	No	No	Not Suggestive of TB	Suggestive of TB	Discordant
1DXXX0003	25/09/2020	Bo	**Acceptable**	**Acceptable**	**No**	**Yes**	No	No	No	No	No	No	Yes	No	No	No	Suggestive of TB	Suggestive of TB	Discordant

EQA: external quality assurance; CXR: Chest-X-ray; TB: Tuberculosis

<u>Supplementary Table S2:</u> Proportion of CXR interpretation discordance per external quality assurance and pre country

Characteristic	Round	Cambodia		Cameroun		Cote d'Ivoire		Mozambique		Sierra Leone		Uganda	
		N	n (%)	N	n (%)	N	n (%)	N	n (%)	N	n (%)	N	n (%)
Airways compression	EQA 1	18	5 (27.7)	12	1 (8.3)	13	0 (0.0)	26	6 (23.0)	48	10 (20.8)	10	0 (0.0)
	EQA 2	7	0 (0.0)	11	1 (9.0)	8	0 (0.0)	9	0 (0.0)	13	1 (7.6)	5	1 (20.0)
	EQA 3	7	0 (0.0)	13	3 (23.0)	24	0 (0.0)			14	3 (21.4)	8	0 (0.0)
	EQA 4			16	3 (18.7)							16	0 (0.0)
Alveolar opacity	EQA 1	18	6 (33.3)	12	3 (25.0)	13	5 (38.4)	26	9 (56.2)	48	12 (25.0)	10	4 (40.0)
	EQA 2	7	0 (0.0)	11	1 (9.0)	8	1 (12.5)	9	4 (44.4)	13	4 (30.7)	5	3 (60.0)
	EQA 3	7	1 (14.2)	13	9 (69.3)	24	0 (0.0)			14	8 (57.1)	8	0 (0.0)
	EQA 4			16	5 (31.2)							16	0 (0.0)
Cavitation	EQA 1	18	2 (11.1)	12	2 (16.6)	13	5 (38.4)	26	4 (15.3)	48	25 (52.0)	10	0 (0.0)
	EQA 2	7	1 (14.2)	11	0 (0.0)	8	2 (25.0)	9	1 (11.1)	13	8 (61.5)	5	1 (20.0)
	EQA 3	7	0 (0.0)	13	1 (7.7)	24	0 (0.0)			14	11 (78.5)	8	0 (0.0)
	EQA 4			16	4 (25.0)							16	0 (0.0)
Enlarged lymph nodes	EQA 1	18	7 (38.8)	12	0 (0.0)	13	7 (53.8)	26	8 (30.7)	48	10 (20.8)	10	2 (20.0)
	EQA 2	7	0 (0.0)	11	0 (0.0)	8	1 (12.5)	9	3 (33.3)	13	5 (38.4)	5	1 (20.0)
	EQA 3	7	3 (42.8)	13	3 (23.0)	24	0 (0.0)			14	6 (42.8)	8	0 (0.0)
	EQA 4			16	6 (37.5)							16	0 (0.0)
Miliary pattern	EQA 1	18	4 (22.2)	12	2 (16.6)	13	0 (0.0)	26	0 (0.0)	48	18 (37.5)	10	1 (10.0)
	EQA 2	7	1 (14.2)	11	0 (0.0)	8	0 (0.0)	9	2 (22.2)	13	5 (38.4)	5	0 (0.0)
	EQA 3	7	2 (28.5)	13	0 (0.0)	24	0 (0.0)			14	3 (21.4)	8	0 (0.0)
	EQA 4			16	0 (0.0)							16	0 (0.0)
Pleural effusion	EQA 1	18	1 (5.5)	12	1 (8.3)	13	1 (7.6)	26	1 (3.8)	48	7 (15.5)	10	0 (0.0)
	EQA 2	7	0 (0.0)	11	0 (0.0)	8	0 (0.0)	9	0 (0.0)	13	2 (15.3)	5	1 (10.0)
	EQA 3	7	0 (0.0)	13	0 (0.0)	24	0 (0.0)			14	4 (28.5)	8	0 (0.0)
	EQA 4			16	1 (6.2)							16	0 (0.0)

EQA: external quality assurance
N: number of CXR read by clinicians and re-read by national reader per country and per EQA;
n (%): proportion of features discordances per country and per EQA

http://dx.doi.org/10.5588/ijtldopen.24.0328

External quality assurance of chest X-ray interpretation to strengthen diagnosis of childhood TB

Supplementary Table S3: Sensitivity and specificity of the chest X-ray interpretation by clinicians against national re-reader's interpretation over external quality assurance per country

Statistic	EQA Round	Cambodia	Cameroon	Cote d'ivoire	Mozambique	Sierra Leone	Uganda
Sensitivity	EQA 1	100 % [3 -100]	100 % [29 - 100]	75 % [19 - 99]	71 % [29 - 96]	100 % [82-100]	50 % [1 - 99]
	EQA 2	100 % [3 -100]	100 % [30 - 100]	100 % [40 - 100]	50 % [7- 93]	100 % [54-100]	50 % [7 - 93]
	EQA 3	100 % [3 -100]	100 % [40 - 100]	100 % [66 - 100]		100 % [72-100]	100 % [54 - 100]
	EQA 4		100 % [54 - 100]				100 % [69 - 100]
Specificity	EQA 1	65% [38 - 86]	56 % [21-86]	33 % [7 - 70]	22 % [3 - 60]	13 % [2 - 40]	50 % [16 - 84]
	EQA 2	100 % [48 - 100]	100 % [63 - 100]	100 % [40 - 100]	40 % [5 - 85]	40 % [5 - 85]	100 % [3 - 100]
	EQA 3	67 % [22 - 96]	11% [0-48]	100 % [66 - 100]		67 % [9 - 99]	100 % [16 - 100]
	EQA 4		50% [16-84]				100 % [48 - 100]

EQA: External Quality Assurance
